# Intraoperative biodegradable stent placement to reduce complications after pancreatoduodenectomy – interim results from a randomised clinical trial

**DOI:** 10.1007/s00423-026-04072-9

**Published:** 2026-05-09

**Authors:** Waqas Farooqui, Jan Henrik Storkholm, Paul Suno Krohn, Luit Penninga, Christian Snitkjaer, Stefan Kobbelgaard Burgdorf

**Affiliations:** 1 Department of Digestive Diseases, Transplantation and Surgery, Rigshospitalet, Copenhagen University Hospital, Copenhagen, Denmark; 2 Department of Surgery, Aalborg University Hospital, Aalborg, Denmark

**Keywords:** Pancreatoduodenectomy, Biodegradable stent, Postoperative pancreatic fistula, Randomised clinical controlled trial

## Abstract

**Introduction:**

Postoperative pancreatic fistula (POPF) remains a major cause of morbidity after pancreatoduodenectomy (PD), with an incidence of more than 30% in patients with small pancreatic ducts. Evidence supporting preventive measures remains limited. Biodegradable stent placement across the pancreaticojejunostomy (PJ) may reduce the POPF. This study represents the first randomised controlled trial evaluating biodegradable stents in patients with high-risk pancreaticojejunostomies.

**Methods:**

This single centre, patient- and assessor-blinded, randomised clinical trial included patients undergoing PD with a main pancreatic duct < 5 mm. Patients were randomised 1:1 to receive a fast-degrading (12-day) ARCHIMEDES biodegradable stent or no stent. The primary endpoint was Clinically relevant postoperative pancreatic fistula CR-POPF. Secondary outcomes included biliary leakage, major complications (Clavien-Dindo ≥ III), length of stay, readmission, and mortality.

**Results:**

In this interim analysis, 50 patients were randomised (26 received a stent, 24 no-stent). Baseline characteristics including Fistula Risk Score (FRS) were comparable. Patients were mainly operated for malignancies. CR-POPF incidence was lower in stent-group compared to no-stent group. However, this difference was not significant (11,50% vs. 25%, *p* = 0,*20*). Biliary leakage and intraoperative blood loss were similar between groups. Two cases of mild, self-limiting postoperative pancreatitis occurred in the stent group. No 30- or 90-day mortality was observed. Median length of stay and readmission rates did not differ significantly.

**Conclusion:**

Interim results from the first blinded, randomised trial with a biodegradable stent, showed no significant reduction in CR-POPF, but a trend towards reducing them. Completing the inclusion and initiating larger multicentre trials are needed to clarify its clinical benefit.

## Introduction

Pancreaticoduodenectomy (PD) is the treatment of choice for malignant and selected benign disorders in the head of the pancreas, the common bile duct, and the ampullary region [1]. Despite improved long-term survival in patients with malignancies, morbidity remains high with incidences up to 50% [2, 3].

Major complications negatively affect long-term survival and quality of life, independent of adjuvant oncological treatment [4]. The most frequent major complications following PD include postoperative pancreatic fistula (POPF), biliary leakage (BL), and postoperative haemorrhage (PPH). The incidence of POPF is 10–12% in high-volume hepatopancreatobiliary (HPB) centres, and is associated with prolonged hospital stay, intensive care unit (ICU) stay, sepsis, and surgical site infections (SSI) [5]. Major complications, particularly POPF and BL affect the patient’s participation in and completion of adjuvant chemotherapy and may reduce disease free survival [6].

The International Study Group of Pancreatic Surgery (ISGPS) classifies POPF according to its impact on the patient’s condition [7]. In a 2016 update on the definition of POPF, the former Grade A was redefined and reformulated as a biochemical leak with fluid accumulation and without the need of intervention. Grade B and C leaks, categorized as clinically relevant POPF (CR-POPF), require drainage, intervention, prolonged ICU stay, or additional surgery.

Surgical risk factors related to POPF, include texture of the pancreas, diameter of the main pancreatic duct (especially diameters smaller than 3 mm), duration of surgery, intraoperative blood loss and need for transfusion, and surgeon or centre experience (high-volume centre versus low-volume centre) [8–11].

The Fistula Risk Score (FRS) is validated to identify patients with a high risk of POPF, and incorporates the texture of the pancreas, pancreatic duct diameter, intraoperative blood loss, and the pathology of the underlying disease [12]. The score stratifies patients into three different risk levels (low, intermediate, and high) based on a calculated score of the above-mentioned variables. The incidence of POPF in patients with several POPF-related risk factors (high FRS) is at least 30% compared to the overall POPF incidence of 10–12% [13, 14]. Patients with a high FRS may require more enhanced postoperative monitoring and potential preventive measures.

Despite extensive research including use of prophylactic somatostatin analogues, fibrin glues and reconstruction techniques, effective measures to prevent POPF remain limited [15, 16]. Internal and external stents in the pancreatojejunostomy have been studied, without significant reductions in POPF, and in some cases even increased the risk of BL, POPF, and other related complications [17, 18].

Biodegradable stents, although novel in PD, appear feasible and safe [19]. A recent systematic review suggested a reduction in POPF, but available studies were few, observational, and lacked comparative groups [20]. However, preliminary evidence supports further evaluation of biodegradable stents in the pancreatojejunostomy.

In this prospective, randomised, blinded clinical trial, we evaluated whether intraoperative placement of biodegradable stents across the pancreatojejunostomy reduced POPF.

## Methods

This is a randomised, parallel-group, patient- and assessor-blinded trial, to evaluate the effect of intraoperative placement of a biodegradable stent across the pancreatojejunostomy (PJ) during PD on postoperative procedure-related complications, with particular focus on POPF.

This trial was conducted at a single, high-volume HPB centre - *Department of Digestive Diseases*,* Transplantation and Surgery*, Rigshospitalet, Copenhagen, Denmark. Our centre performs an average of 200 pancreatic resections annually. The same three senior, attending HPB-surgeons performed the procedures.

### Sample size justification

The primary endpoint of this randomized controlled trial is the occurrence of CR-POPF. Based on published data, the incidence of CR-POPF in patients undergoing high-risk pancreatojejunostomy was assumed to be at least 30% [13, 14]. The intervention was hypothesized to result in a 50% relative reduction in CR-POPF.

Sample size calculation was performed for a two-sided comparison, with a significance level *(α)* of 0.05 and a power *(β)* of 80%. The total number of patients requires in each group was 121 patients, to detect a 50% reduction in POPF. To account for potential dropouts, protocol deviations and interim analyses, the sample size was increased slightly, resulting in a total sample size of 310 patients (155 per group).

A sample size of 50 patients was determined for interim analyses of feasibility and safety. Based on the average PD procedures over a year, at our high-volume HPB centre, inclusion of 50 eligible patients would approximately take 15 months.

### Randomization, allocation concealment and blinding

Patients were randomised following a 1:1 ratio to either the intervention group (stent) or the control group (no-stent). The randomisation was performed by contacting an independent research unit and using a web-based tool with a computer-generated allocation sequence list (Clinical Trial Randomization Tool, National Cancer Institute, https://ctrandomization.cancer.gov). The allocation sequence list was known only by the research unit and concealed from investigators until the trial database was closed, data analysed, and the abstract for the trial report was written. Outcome assessors, the trial statistician, and the lead author were blinded to group allocations, which was coded as 0 and 1. Due to the nature of the intervention and control, blinding of the surgeons performing the procedures was not possible.

### Inclusion criteria

Patients were included consecutively during the inclusion period, and were included if they met all the following criteria:

1. Undergoing a pancreatoduodenectomy.
2. Main pancreatic duct diameter <5 mm, assessed on a computed tomography scan (CT scan) or magnetic resonance imaging scan (MRI scan) and re-assessed intraoperatively.
3. Age ≥ 18 years.
4. Written informed consent provided.

### Exclusion criteria

Patients were excluded if they met any of the following criteria:

1. Inability to provide informed consent.
2. Previous surgery on the bile ducts, including ERC or PTC-assisted drainage.
3. High risk of, or history of, multiple thromboembolic or bleeding disorders.
4. Ongoing immunosuppressive therapy.

### Trial interventions

PD was performed as an open procedure, with an “artery-first” approach, en bloc with prepyloric amputation, resection of the pancreatic head, duodenum, and gallbladder. The division of the common hepatic duct was done above the cystic duct, just below the confluence. Reconstruction was performed with retrocolic pancreatojejunostomy, hepaticojejunostomy, and antecolic gastrojejunostomy, with all anastomoses on the same segment of the jejunum. After performing the resection, the construction of the pancreatojejunostomy was initiated. Depending on duct diameter and pancreatic texture, the pancreatojejunostomy was constructed using either the Blumgart technique [21] or the classic technique [22] This was independent of the group allocation.

To maintain a homogenous high-risk cohort in accordance with the known risk factors for POPF, if the patient met the inclusion criteria and the pancreas had a small duct diameter (< 5 mm), a 6 Fr gauge, fast-degrading (12 days) ARCHIMEDES biodegradable pancreatobiliary stent (amg International GmbH, Winsen, Germany) was inserted into the anastomosis before completion. No sutures were used to anchor the stent.

Intraoperative blood loss was measured by weighing surgical gauzes infused with blood and measured blood loss accumulated during intraoperative suction. If blood transfusions were needed, this was registered as well, as number of packed red blood-cell units used.

The control group underwent PD without stent placement.

All patients followed the standard postoperative care protocol in our institution. This included placement of an intraabdominal drain behind the pancreatojejunostomy and hepaticojejunostomy. Corticosteroids, somatostatin analogues or other researched prophylactic measures are not used during standard perioperative care. As a part of our standard care, drain fluid amylase was measured on postoperative days three and four with drain removal if values were within the normal range (less than three times the upper limit of normal serum-amylase) and a low volume. Antibiotics were administered intravenously until postoperative day three and prolonged according to the clinical development. Oral intake was allowed after surgery, gradually increasing the consistency from water to solid depending on the patient’s tolerance. An epidural was placed to alleviate postoperative pain and was substituted with oral or transdermal analgesics the fourth postoperative day.

#### Primary outcome measures

- Incidence of CR-POPF, defined by the ISGPS criteria [7].

## Secondary outcome measures

- Overall incidence of complications after PD, classified according to Clavien-Dindo [23].
- Biliary leakage defined by ISGPS criteria [24].
- 30-day and 90-day mortality.
- Length of hospital stay (LOS).
- 30-day readmission rate.
- Estimated intraoperative blood loss (EBL).
- Drain amylase levels on postoperative day three and four.

### Statistical analysis

Continuous variables were reported as medians with interquartile range (IQR) or means with standard deviations (SD), as appropriate, and compared using the Mann-Whitney U test. Categorical variables were compared using X^2^ (chi-square). Univariate and multivariate logistic regression analyses were used to identify independent risk factors. Post hoc analyses were performed to explore differences in clinically relevant subgroups. Statistical analyses were conducted using SPSS version 26 (SPSS Inc., Chicago, Illinois, USA) for Windows (Microsoft Corporation, Redmond, Washington, USA).

### Trial ethics and conduct

This trial adheres to the CONSORT guidelines [25], the latest version of the Helsinki Declaration [26], and national Danish legislation. The trial was registered at ClinicalTrials.gov (NCT06205693) before patient inclusion commenced. The study protocol, patient consent form, and information sheet were approved by the Capitol Region Committee on Health Research Ethics and the regional branch of the Danish Data Protection Agency (H-22070655). All data were stored and protected in accordance with the Data Protection Act and Data Protection Regulations. Informed consent was obtained from all participants prior to inclusion.

## Results

**Fig. 1:**
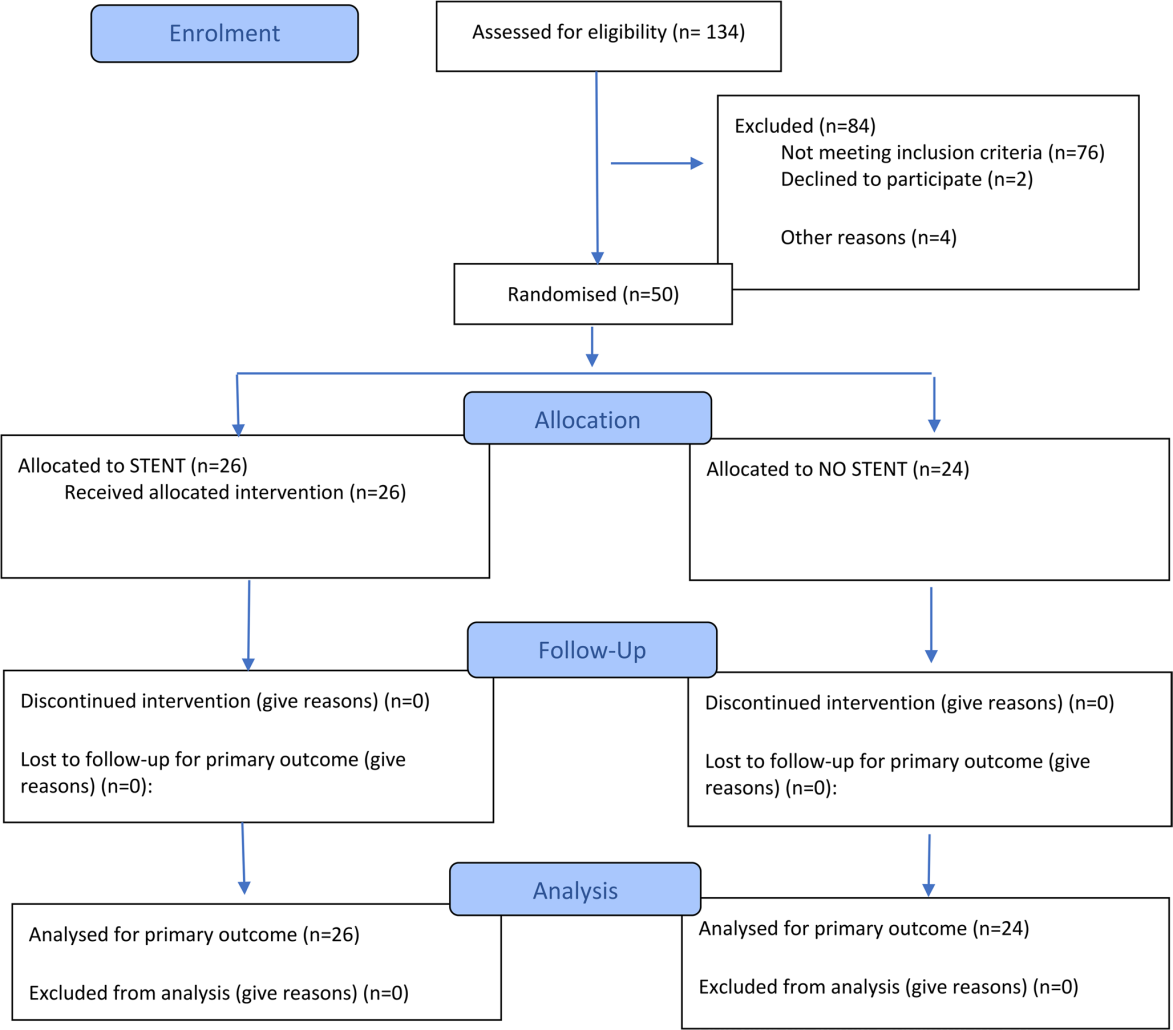
CONSORT 2025 Flow Diagram. Flow diagram of the progress through the phases of a randomised trial of the two groups (Stent and No-stent)

Patients were included from October 1 st, 2023, until January 1 st, 2025. In this period, a total of 50 patients undergoing pancreatoduodenectomy were included, with 26 allocated to the stent group and 24 patients to the no-stent group. The CONSORT diagram [25] is presented in Fig. 1.

Baseline characteristics were comparable between groups (Table 1). There were no significant differences in age sex distribution, BMI, or ASA-score. The mean FRS was 6,9 and 7,0 in the stent group and no-stent group (*p = 0*,*98)*.

**Table 1:** Baseline characteristics

	Group 1 (Stent), *n* = 26	Group 2 (No Stent), *n* = 24
Age, years	67.50 (55.25–76.50)	67.00 (59.75–72.00)
Sex, male (%)	16 (61.50)	17 (70.10)
BMI, (IQR)	27.20 (23.80–28.90)	23.90 (22.60–26.10)
ASA-score (%)		
1	4 (15.40)	2 (8.30)
2	14 (53.80)	18 (75.00)
3	5 (30.80)	4 (16.70)
Fistula Risk Score, points	6.8 (4–9)	7.0 (5–9)
Pancreaticojejunostomy, %		
Blumgart	21 (80.80)	19 (79.20)
Classic	5 (19.20)	5 (20.80)
Malignant pathology, %	21 (95.30)	18 (81.80)
PDAC, n	8	6
Papillary, n	5	2
Duodenal ADC, n	6	5
dCC, n	3	6
NET, n	1	1

Numerical values presented as median, IQR unless stated otherwise. BMI: Body Mass Index; ASA: American Society of Anesthesiologists Physical Status Classification System; PDAC: Pancreatic ductal adenocarcinoma; ADC: Adenocarcinoma; dCC: Distal cholangiocarcinoma; NET: Neuroendocrine tumour

The incidence of CR-POPF did not differ significantly − 11.5% in the stent group versus 25.0% in the no-stent group (*p = 0.20*). Drain amylase levels on the third and fourth postoperative day did not differ significantly between the groups.

Median intraoperative blood loss was 1120mL (IQR 806–1725) in the stent group and 1055mL (IQR 876–1255) in the no-stent group (*p = 0.58*).

Major postoperative complications (≥ CD3) occurred in 30.8% of stented patients and 37.5% of non-stented (*p = 0.80*). Biliary leakage occurred in 15.4% and 16.7% of patients in the stent and no-stent groups, respectively (*p = 1.0*). Two patients in the stent group developed postoperative pancreatitis in the pancreas remnant; no similar cases in the no-stent group - the incidence was not significant.

The median length of stay was 12.0 days in the stent group and 18.0 days in the no-stent group (*p = 0.48*). Readmission within 30 days occurred in 28.6% and 36.4% of patients, respectively (*p = 0.82*).

No deaths occurred within 30 or 90 days.

An overview of primary and secondary outcomes is present in Table 2.

**Table 2:** Primary and secondary outcomes

	Group 1 (Stent), *n* = 26	Group 2 (No Stent), *n* = 24	RR (95% CI)	*P*-value
Intraoperative bleeding, mL (IQR)	1120 (806.25–1725.00)	1055 (876.25–1255.00)		0.58
Drain-amylase, U/L, median (range)				
POD 3	290 (9–13200)	209.5 (10–2760)		0.62
POD 4	61.5 (6–24700)	52.5 (5–6540)		0.90
Leakage of PJ	3 (11.50)	9 (37.50)	0.31 (0.09–1.00.09.00)	0.03
Biochemical Leak	0 (0)	3 (12.50)		0.01
Leakage of PJ (CR-POPF)	3 (11.50)	6 (25.00)	0.46 (0.13–1.64)	0.20
*Grade B*	*1 (4.30)*	*5 (20.80)*		*0.19*
*Grade C*	*2 (8.60)*	*1 (4.30)*		*0.62*
Leakage of HJ	4 (15.40)	4 (16.70)	0.92 (0.26–3.29)	1.00
Clavien Dindo				
< CD3	7 (27.00)	6 (25.00)	1.08 (0.42–2.79)	0.90
≥CD3	8 (30.80)	9 (37.50)	0.82 (0.36–1.86)	0.80
Length of stay, days	12.00 (9.00–22.30.00.30)	18.00 (8.75–28.75)		0.48
Readmission within 30 days	6.00 (28.60)	8.00 (36.40)	0,69 (0.28–1.71)	0.82
30-day mortality, %	0 (0)	0 (0)		NA
90-day mortality, %	0 (0)	0 (0)		NA
Adjuvating oncological treatment	12 (46.20%)	15 (62.50%)		0.44
Time from operation to adjuvant oncological therapy (days, IQR)	48 (42.00–62.00)	65.50 (55.50–77.00)		0.06

Numerical values presented as median, IQR unless stated otherwise. For binary outcome, Effect estimates are presented as Risk Ratio (RR) and 95% Confidence Intervals (CI). POD: Postoperative Day; POPF: Postoperative Pancreatic Fistula; PJ: Pancreaticojejunostomy; HJ: Hepaticojejunostomy; CR: Clinical Relevant; CD: Clavien-Dindo; NA: Not Applicable

## Discussion

This study is the first randomised clinical trial assessing the efficacy of biodegradable stents across the PJ, on the incidence of POPF. Intervention with biodegradable stents was not associated with a significant reduction in CR-POPF; however, there was a trend towards the stent reducing the incidence of CR-POPF. Baseline characteristics, underlying pathology, and surgical factors including FRS, were comparable between groups, suggesting that the observed reduction in POPF was likely attributable to the intervention itself rather than confounding variables. The intervention was technically feasible, and although we observed two cases of pancreatitis in the stent group.

The patients in the stent group who developed postoperative pancreatitis in the pancreatic remnant, were mild and self-limiting but deserve consideration. Potential contributing factors include local irritation or transient obstruction caused by the stent, particularly in patients with very small ducts (< 3 mm). Stent placement technique – placing the stent in the pancreatic duct first before placing it in the jejunal limb or vice versa, or deep insertion of the stent into the remnant, could cause excessive manipulation of the pancreatic duct, provoking local inflammation. Temporary obstruction of side-branches by degradation fragments may also have contributed, and refinements in stent design or placement could mitigate these risks in future studies.

A total of nine patients experienced CR-POPF. Three in the stent group (one Grade B, and two Grade C) and six in the no-stent group (five Grade B and one Grade C). What was common for these patients was their length of stay were all well over 20 days, with the longest admission time being 70 days and upon discharge. POPF was primarily discovered after removing the surgical drain (on POD 5), with normal drain amylase levels on POD 3 and POD 4. Patients were mainly treated with percutaneous drainage of fluid accumulations. The three patients with Grade C POPF were reoperated. One underwent rescue total pancreatectomy on POD 2, one had surgical drainage of the intraabdominal fluid accumulations on POD 20, after numerous attempts with percutaneous drainage. The last patient had a fascia dehiscence on POD 5, which was repaired, and during this procedure, an anterior dehiscence of the PJ was observed and attempted to repair, by the non-HPB, attending surgeon on call. Upon discharge only three of the nine patients managed to start adjuvant chemotherapy with the earliest starting 54 days after surgery. Major complications after PD, including POPF, negatively affect long-term survival and quality of life, independent of adjuvant oncological treatment [4]. Completing adjuvant chemotherapy prolongs recurrence free survival but POPF affects the patient’s participation in and completion of adjuvant chemotherapy [6] as we also experienced in this trial.

The pathophysiology of POPF is yet to be fully understood. Traditionally, POPF has been attributed to surgical failure of the PJ, prompting numerous studies on anastomotic techniques, emphasising proper and adequate suturing and approximation of the mucosal layers as paramount [27]. Likewise, assuring vascularity of the pancreas, especially at the site of the anastomosis, is also important for healing [28, 29].

Besides refining the surgical techniques, other prophylactic methods including non-degradable stents, whether internal or external, have also been investigated, though with concerns of their potential to increase POPF in certain cases [30]. Various other strategies to reinforce the PJ – such as sealants, patches or wrapping the anastomosis with omentum or the falciform ligament – have been tested, but none have demonstrated consistent benefits [27].

Although not a direct cause, leakage of enzyme-rich pancreatic secretions into the peritoneal cavity is believed to exacerbate POPF via inflammation, tissue injury, and delayed anastomotic healing. Somatostatin analogues are thought to reduce this risk by decreasing pancreatic exocrine secretion [31].

Despite numerous attempts, no single strategy has been shown to significantly reduce the incidence of CR-POPF [32].

The rationale for stenting across the PJ is to provide decompression and physical stability, minimizing misalignments of the mucosal layers and maintaining structural integrity of pancreatic duct and anastomosis during the critical phase of tissue healing. Biodegradable stents differ from conventional non-degradable stents in both material and clinical behaviour. Traditional stents remain permanently, and may cause obstruction, ductal irritation, infection, or strictures. In contrast, the biodegradable stent undergoes hydrolytic degradation, providing temporary decompression of the PJ without the long-term presence of a foreign body. Also, traditional stents are tubular and non-fixated, which can result in stent migration [33]. This biodegradable stent (ARCHIMEDES, amg International GmbH, Winsen, Germany) has a double-helical structure and self-fixating, designed to remain in place without sutures, reducing the risk of migration. The double-helix design is meant to allow the fluid to flow along the stent rather than through a lumen, which theoretically reduces the risk of clotting.

Most studies on POPF focus on CR-POPF, as these are associated with major complications such as prolonged hospitalization, ICU admission, and reoperation. Biochemical leaks are typically self-limiting and thus receive less emphasis. Biochemical leak cannot be reclassified as a CR-POPF, since the classification is defined retrospectively. However, early subclinical leaks may precede clinically relevant fistulas, in up to 30% of cases [34, 35]. Reducing early biochemical leaks, as was the case in our trial, may therefore lower the overall incidence of CR-POPF.

The use of this biodegradable stent was feasible regardless of the PJ reconstruction technique, including both the Blumgart and classic PJ techniques. Comparable outcomes between techniques suggest that the protective effect of the stent is independent of the anastomotic approach.

This trial has some limitations. The single-centre design and modest sample size limit statistical power and may have prevented the detection of a significant reduction in CR-POPF. Large multicentre trials, ideally stratified by fistula-risk scores and anastomotic technique, are needed to determine whether biodegradable stents meaningfully reduce CR-POPF. Our centre intends to continue the trial, with a greater cohort based on the sample size calculation, hoping to involve other high-volume HPB-centers. An ongoing randomized trial (ClinicalTrials NCT05668260) and future pooled analyses may provide more definitive evidence. Also, due to the surgical standards at our centre, our study was only conducted on PJ and not on pancreatogastrostomies (PG). Some trials conclude PG being superior to PJ in high-risk pancreatic anastomoses [36]. Trials comparing non-degradable stent placement in a PG have been conducted previously [37, 38], and future multicentre trials could consider stratifying according to these anastomotic techniques too.

Computed tomography imaging to assess stent position or degradation was not performed to maintain blinding and because the stent’s expected degradation time (12 days) made later imaging unlikely to yield meaningful information.

In summary, after randomising 50 patients, intraoperative placement of this biodegradable stent was not associated with a lower rate of CR-POPF, but there was a non-significant trend towards lower CR-POPF. Completing enrolment for this trial and initiating future multicentre trials with larger sample sizes, stratification by fistula risk, and comprehensive assessment of efficacy are warranted.

## Data Availability

The authors have no conflicts of interest. Data is available upon request to the corresponding author.
